# New insights into the post-translational modification of multiple phosphoenolpyruvate carboxylase isoenzymes by phosphorylation and monoubiquitination during sorghum seed development and germination

**DOI:** 10.1093/jxb/erw186

**Published:** 2016-05-18

**Authors:** Isabel Ruiz-Ballesta, Guillermo Baena, Jacinto Gandullo, Liqun Wang, Yi-Min She, William Charles Plaxton, Cristina Echevarría

**Affiliations:** ^1^Departamento de Biología Vegetal, Facultad de Biología, Universidad de Sevilla, Avda Reina Mercedes nº 6, 41012 Sevilla, Spain; ^2^Shanghai Center for Plant Stress Biology, Chinese Academy of Sciences, 3888 Chenhua Road, Shanghai 201602, China; ^3^State Key Laboratory of Crop Genetics and Germplasm Enhancement, Soybean Research Institute, Nanjing Agricultural University, Nanjing, Jiangsu 210095, China; ^4^Department of Biology, Queen’s University, Kingston, Ontario K7L 3N6, Canada

**Keywords:** Germination, monoubiquitination, phosphorylation, phospho*enol*pyruvate carboxylase, post-translational modifications, seed development, seeds, *Sorghum bicolor*, tissue-specific gene expression.

## Abstract

Phosphoenolpyruvate carboxylase (PEPC; E.C. 4.1.1.31) was characterized in developing and germinating sorghum seeds, focusing on the transcript and polypeptide abundance of multiple *plant-type phosphoenolpyruvate carboxylase* (*PTPC*) genes, and the post-translational modification of each isoenzyme by phosphorylation versus monoubiquitination during germination. We observed high levels of *SbPPC4* (*Sb07g014960*) transcripts during early development (stage I), and extensive transcript abundance of *SbPPC2* (*Sb02g021090*) and *SbPPC3* (*Sb04g008720*) throughout the entire life cycle of the seed. Although tandem mass spectrometry (MS) analysis of immunopurified PTPC indicated that four different PTPC isoenzymes were expressed in the developing and germinating seeds, SbPPC3 was the most abundant isozyme of the developing seed, and of the embryo and the aleurone layer of germinating seeds. *In vivo* phosphorylation of the different PTPC isoenzymes at their conserved N-terminal seryl phosphorylation site during germination was also established by MS/MS analysis. Furthermore, three of the four isoenzymes were partially monoubiquitinated, with MS/MS pinpointing SbPPC2 and SbPPC3 monoubiquitination at the conserved Lys-630 and Lys-624 residues, respectively. Our results demonstrate that monoubiquitination and phosphorylation simultaneously occur *in vivo* with different PTPC isozymes during seed germination. In addition, we show that PTPC monoubiquitination in germinating sorghum seeds always increases at stage II (emergence of the radicle), is maintained during the aerobic period of rapid cell division and reserve mobilization, and remains relatively constant until stage IV–V when coleoptiles initiate the formation of the photosynthetic tissues.

## Highlight

This work documents the remarkable versatility and complexity of *in vivo* post-translational modifications (monoubiquitination and phosphorylation) of different plant-type phosphoenolpyruvate carboxylase (PEPC) isoenzymes during the life cycle of sorghum seeds.

## Introduction

Phosphoenolpyruvate (PEP) carboxylase (PEPC; EC 4.1.1.31) is an important, tighly regulated, cytosolic metabolic enzyme that plays a pivotal photosynthetic role in primary CO_2_ fixation by C_4_ and Crassulacean acid metabolism plants, but also has a variety of additional functions, including roles in seed development and germination (O’Leary *et al.*, 2011*a*). It catalyses the irreversible β-carboxylation of PEP to form oxaloacetate (OAA) and orthophosphate (P_i_) (Chollet *et al*., 2006). The *plant-type PEPC* (*PTPC*) genes encode closely related 100–110kDa polypeptides that typically exist as Class-1 PEPC tetramers that are post-translationally regulated by a combination of several allosteric effectors, including Glc-6-P (activation) and L-malate (inhibition), reversible phosphorylation (Nimmo, 2003; O’Leary *et al.*, 2011*a*), and monoubiquitination (Uhrig *et al.*, 2008, Shane *et al.*, 2013; Ruiz-Ballesta *et al.*, 2014). The metabolite control is integrated with the enzyme’s reversible phosphorylation at a highly conserved serine residue located near the N-terminus of its PTPC subunits (Chollet *et al.*, 1996; Echevarría and Vidal, 2003; O’Leary *et al.*, 2011*a*). Phosphorylation activates the enzyme by making it less sensitive to feedback inhibition by malate. Phosphorylation is catalysed by a PTPC-kinase (more commonly referred to as PEPC-kinase; PPCK), whereas dephosphorylation is catalysed by a protein phosphatase type-2A (Nimmo, 2000; Izui *et al.*, 2004; O’Leary *et al.*, 2011*a*). Both controls are interactive and thus allow a fine modulation of the enzyme’s kinetic properties as required by the physiological context (Echevarría and Vidal, 2003). Regulatory PTPC monoubiquitination has been demonstrated in the endosperm of germinating castor oil seeds (COS) (Uhrig *et al.*, 2008), in developing proteoid roots of P-deficient harsh hakea (Shane *et al.*, 2013), and in lily pollen (Igawa *et al.*, 2010). A recent study by our group has shown monoubiquitination of SbPPC3 in sorghum, a cereal with starch-storing seeds (Ruiz-Ballesta *et al.*, 2014). Monoubiquitination is inhibitory as it results in increased *K*_m_ (PEP) values and enhanced sensitivity to allosteric inhibitors (Uhrig *et al.*, 2008; Shane *et al.*, 2013; Ruiz-Ballesta *et al.*, 2014). In COS, PTPC monoubiquitination and phosphorylation appear to be mutually exclusive, while PTPC polypeptides are alternatively phosphorylated or monoubiquitinated in developing proteoid roots of harsh hakea (Shane *et al.*, 2013). In addition, the elimination of photosynthate supply caused by depodding in COS triggers *in vivo* dephosphorylation and subsequent partial monoubiquitination of PTPC polypeptides (Uhrig *et al.*, 2008; O’Leary *et al.*, 2011*b*). Previous studies have indicated that PTPC polypeptides become *in vivo* phosphorylated in de-embryonated sorghum seed during imbibition (i.e. on the basis of a significant increase in the enzyme’s *IC*_50_ for L-malate and *in vivo* ^32^P incorporation) (Nhiri *et al.*, 2000). In other starch-storing cereal seeds such as barley and wheat, the phosphorylation of both PTPC polypeptides during germination has also been confirmed by immunoprecipitation, followed by SDS-PAGE and autoradiography of the *in vivo* ^32^P-labeled PEPC (Osuna *et al.*, 1999; Feria *et al.*, 2008). As far as we know, no previous study has focused on how these post-translational modifications (PTMs) are combined in the different subunits and isoenzymes of a PEPC’s protein family. Deciphering how monoubiquitination and phosphorylation influence each PTPC isoenzyme during the life cycle of sorghum seeds was a major objective of the current study.

*Bacterial-type PEPC* (*BTPC*) genes encode larger 116–118kDa polypeptides exhibiting low (<40%) sequence identity with PTPCs. BTPC lacks the conserved N-terminal seryl phosphorylation motif characteristic of PTPCs, and in vascular plants it appears to only exist as catalytic and regulatory subunits of novel Class-2 PEPC heteromeric complexes composed of tightly associated PTPC and BTPC subunits (Igawa *et al.*, 2010; O’Leary *et al.*, 2011*a*; Park *et al.*, 2012). Using western blot analysis and specific anti-BTPC-IgGs in crude extract of barley seeds, Class-2 PEPC complexes have not been identified (Feria *et al.*, 2008). This is an interesting finding as the BTPC and Class-2 PEPC appear to play an important role during COS development (Uhrig *et al.*, 2008). Assessing, by MS/MS and qRT-PCR, the possible occurrence of BTPC (SbPPC6) and/or Class-2 PEPC during the course of the life cycle of sorghum seeds was also an important objective of this work.

Sorghum (*Sorghum bicolor* L. Moench) is the world’s fifth most important cereal crop (www.fao.org) and provides food, feed, fiber, fuel, and chemical/biofuel feedstocks across a range of environments and production systems (Kresovich *et al.*, 2005). Sorghum encodes five *PTPC* genes (Paterson *et al.*, 2009): *Sb02g021090* and *Sb04g008720* encode housekeeping and inducible SbPPC2 and SbPPC3, respectively (Lepiniec *et al.*, 1993), whilst *Sb07g014960* and *Sb03g035090* encode PTPC isoenzymes SbPPC4 and SbPPC5 but these have unknown functions. All of these are C_3_-type PTPCs. *Sb10g021330* encodes C_4_ photosynthetic SbPPC1 subunits that are highly expressed in sorghum leaves. In addition, *Sb03g008410* (*SbPPC6*) encodes a single BTPC (Table 1).

**Table 1.** The sorghum *PEPC* gene family. Names used in this work for the different *PEPC* genes, the equivalence with other names, and assigned functions described by Lepiniec et al. (1993, 1994), and orthologs established by Paterson et al. (2009). Sb, Sorghum bicolor; Sv, Sorghum vulgare; Zm, Zea mays; Os Oryza sativa.

**Name of gene**	**Accession number** ^**a**^	**Named by** Lepiniec *et al.* (1993 **) and** Ruiz-Ballesta *et al.* (2014)	**Known function**	**Orthologs** (Paterson *et al.*, 2009)
*SbPPC1*	Sb10g021330	CP46	C_4_ PTPC , photosynthetic C_4_ gene	X63756 (SvPEPC1); NM001111948 (ZmPEPC1)
*SbPPC2*	Sb02g021090	CP28	C_3_ PTPC, housekeeping gene	X59925 (SvPEPC4); NM001111968 (ZmPEPC2)
*SbPPC3*	Sb04g008720	CP21	C_3_ PTPC, root-inducible gene	X65137 (SvPEPC3); NM001112033 (ZmPEPC3
*SbPPC4*	Sb07g014960	-	C_3_ PTPC, unknown so far	Os08g0366000
*SbPPC5*	Sb03g035090	-	C_3_ PTPC, unknown so far	Os01g0758300
*SbPPC6*	Sb03g008410	-	BTPC, as Class-2 PEPC	Os01g0110700
a NCBI database				

In this study, the biochemical and immunological properties of PTPCs during development and germination of starch-storing sorghum seeds were further characterized. We also elucidated the transcript abundance of *PTPC* genes, and determined subunit composition, relative abundance, and PTMs of each PTPC isoenzyme during germination. As far as we know, this represents the most detailed study to date that examines both phosphorylation and monoubiquitination of a PTPC protein family in any plant species. Furthermore, we show that enhanced PTPC monoubiquitination always accompanies radical emergence during seed germination, and that both processes are accelerated in parallel under aerobic conditions or elevated temperature.

## Materials and methods

### Plant material

Mature sorghum [*Sorghum bicolor* (L.) Moench, var. PR87G57 and var. PR88Y20, Pioneer Hi-Bred, Spain] seeds were sterilized and germinated for up to 96h at 25 or 35 ºC as previously reported (Nhiri *et al.*, 2000). For immunological studies or to analyze PEPC activity, the aleurone layers, starchy endosperm (aleurone-endosperm), and embryos were carefully dissected at the indicated times post-imbibition. Sterilized sorghum seeds were also submerged in distilled water (hypoxic condition) or placed on moistened filter paper (normoxic condition) at 25 ^o^C for 48 and 72h. Harvested tissues were frozen in liquid nitrogen and stored at –80 ºC until used. Developing seeds were obtained from sorghum plants cultivated in a greenhouse under a 14h day (25 ºC)/10-h night (18 ºC) cycle. Seeds were harvested at various stages of development, ground to a powder under liquid nitrogen, lyophilized, and stored at 4 ºC for later analyses.

### Antibodies

Polyclonal antibodies against native C_4_-photosynthetic SbPPC1 from sorghum leaves (anti-C_4_ PTPC) were prepared as described in Pacquit *et al.* (1995).

Anti-C19 and anti-N24 were raised, respectively, against synthetic peptides corresponding to the C-terminal [(Y) EDTLILTMK GIAAGMQNTG] and the dephosphorylated N-terminal [ERHHSIDAQLRALAPGKVSEE24(YG)] ends of SbPPC1 (i.e., sorghum C_4_-photosynthetic PTPC) as previously described (Ruiz-Ballesta *et al.*, 2014). Anti-COS PTPC was raised against native Class-1 PEPC purified from the endosperm of developing castor oil seeds as described in Tripodi *et al.* (2005).

### Immunohistochemistry

Seeds germinated at 24h post-imbibition were fixed in 4% (w/v) paraformaldehyde and in 0.25% (v/v) glutaraldehyde dehydrated in a graded series of aqueous ethanol solutions, and embedded in Paraplast Plus (Panreac) as described by González *et al.* (1998). Sections (15 µm thick) were prepared using a microtome (model RM2165, Leica) and placed on poly-L-Lys-coated microscope slides. After deparaffinizing in xylol and rehydrating in decreasing concentrations of ethanol, sections were blocked for 30min in TBS buffer containing 3% (w/v) BSA. Then, 500 µl of the same solution containing affinity-purified anti-C_4_ PTPC (3 µg of protein) (Pacquit *et al.*, 1995) or pre-immune serum was placed on the samples and incubated overnight at 4 °C. Unbound antibodies were removed by three 10-min washes in TBS. Tissue sections were then incubated with alkaline phosphatase-conjugated goat anti-(rabbit IgG)-IgG (Bio-Rad) for 2h at 37 °C. The reaction of alkaline phosphatase was developed colorimetrically with a BCIP/NBT Liquid Substrate System (Sigma).

### Protein extraction

Frozen powder (0.4g) from whole developing or germinated sorghum seeds was ground in a chilled mortar with sand in 1ml of an ice-cold buffer that contained 100mM Tris-HCl (pH 7.5), 10mM MgCl_2_, 1mM EDTA, 20% (v/v) glycerol, 14mM β-mercaptoethanol, 1mM phenylmethylsulfonyl fluoride, 10 µg ml^−1^ chymostatin, 10 µM leupeptin, 50mM KF, 1mM Na_2_MoO_4_, 1mM Na_3_VO_4_^−^, and 50nM microcystin-LR. Homogenates were centrifuged at 17000 *g* for 7min and the supernatants (0.5ml) desalted through 3-ml Sephadex G-25 spin columns equilibrated in extraction buffer (lacking PVPP and PVP) prior to enzymatic analysis.

### Electrophoresis and immunoblotting

SDS-PAGE, non-denaturing PAGE and in-gel PEPC activity staining, immunoblotting, and visualization of antigenic polypeptides using an alkaline-phosphatase-conjugated secondary antibody with chromogenic detection were performed as previously described (Rivoal *et al.*, 2002; Tripodi *et al.*, 2005). Alternatively, immunoreactive polypeptides were detected using a chemiluminescence system (SuperSignal West Pico Rabbit IgGs; Thermo Scientific) according to the manufacturer’s instructions in a Fujifilm LAS3000 mini-system. The immunoreactive polypeptides were quantified via analysis of the scanned blots using Multi-Gauge V3.0 software (Fujifilm).

### Determination of PEPC activity and protein concentration

PEPC activity was assayed at 340nm using a Molecular Devices Spectramax Kinetics Microplate reader. Optimized assay conditions were: 50mM HEPES-KOH (pH 8.0), 2.5mM PEP, 5mM NaHCO_3_, 5mM MgCl_2_, 0.15mM NADH, 10% (v/v) glycerol, 1mM DTT, and 5 units ml^−1^ porcine heart malate dehydrogenase (0.2ml final volume). One unit of activity is deﬁned as the amount of PEPC catalysing the production of 1 µmol of oxaloacetate min^−1^ at 25 ºC. Protein concentrations were determined by the Coomassie Blue G-250 dye-binding method using bovine γ-globulin as the standard (Tripodi *et al.*, 2005).

### Immunoprecipitation and immunoaffinity chromatography

Immunoprecipitation of PTPC polypeptides from clarified seed extracts was performed using anti-C_4_ PTPC as previously described (Osuna *et al.*, 1999).

For immunoaffinity chromatography, frozen whole 48-h germinated sorghum seeds (5g) were homogenized (1:2.5; w/v) using a Polytron PT-3100 homogenizer in ice-cold buffer A that contained 100mM HEPES-KOH (pH 7.5), 1mM EDTA, 1mM EGTA, 15% (v/v) glycerol, 5mM MgCl_2_,100mM KCl, 10mM NaCl, 25mM NaF, 1mM Na_3_VO_4_^−^, 0.1% (w/v) polyvinylpolypyrrolidone, 0.1% (v/v) Triton X-100, 2mM 2,2′-dipyridyl disulfide, and 10 µl ml^−1^ ProteCEASE-100 (G-BioSciences). Homogenates were centrifuged at 4 ºC for 10min at 48500 *g*, and the supernatant fluid re-centrifuged for 5min and filtered through a layer of Miracloth (Calbiochem). Clarified extracts were pre-cleared at 25 ºC by eluting at 0.5ml min^−1^ through an AminoLink column (1×4cm) that had been pre-saturated with 1M Tris-HCl (pH 7.4). Unbound proteins were immediately absorbed at 1ml min^−1^ onto an anti-COS PTPC immunoaffinity column (1×2cm; prepared as described by Uhrig *et al.*, 2008) that had been pre-equilibrated with PBS. Flow-through fractions were collected by eluting the column at 1ml min^−1^ with PBS until *A*_280_ decreased to baseline, and bound proteins were eluted at 0.5ml min^−1^ with 100mM Gly-HCl (pH 2.8), and immediately neutralized (1-ml fractions collected into 0.1ml of unbuffered 1M Tris). *A*_280_ absorbing fractions were concentrated to ~1mg ml^−1^ and analyzed for their polypeptide composition by SDS-PAGE and immunoblotting.

### Mass spectrometry

In the initial MALDI-TOF MS analysis, PTPCs at the different stages of seed development and germination were extracted and purified by immunoprecipitation from the clarified extracts, followed by SDS-PAGE of the solubilized immunoprecipitates (Osuna *et al.*, 1999). Protein-staining bands of interest were manually excised from micro-preparative gels using biopsy punches, then reduced, alkylated, and digested with trypsin according to Sechi and Chait (1998). Briefly, gel bands were washed twice with water, shrunk for 15min with 100% acetonitrile, and dried in a Savant SpeedVac for 30min. Then the samples were reduced with 10mM dithioerythritol in 25mM ammonium bicarbonate (NH_4_HCO_3_) for 30min at 56 ºC and subsequently alkylated with 55mM iodoacetamide in 25mM NH_4_HCO_3_ for 15min in the dark. Finally, samples were digested with 12.5ng µl^−1^ sequencing grade trypsin (Roche Molecular Biochemicals) in 25mM NH_4_HCO_3_ (pH 7.6) overnight at 37 ºC. After digestion, 1 µl of each sample was spotted onto a MALDI plate and allowed to air-dry at room temperature. Then, 0.4 µl of 3mg ml^−1^ α-cyano-4-hydroxy-cinnamic acid as the matrix (Sigma) in 50% acetonitrile was added onto the spots and they were again allowed to air-dry at room temperature.

Sample analyses were performed on a 4800 Plus Proteomics Analyzer MALDI-TOF/TOF mass spectrometer (AB Sciex, Toronto, Canada). The instrument was operated in the positive reflector mode with an accelerating voltage of 20 000V. Mass spectra were calibrated internally using the peptides of trypsin autolysis. Mass spectrometric measurements yielded a list of monoisotopic masses of the observed peptides at a signal-to-noise ratio greater than 10, which were used for peptide mapping.

Peptides of interest were further analyzed by MS/MS in order to assess their sequences. Suitable precursor ions were selected from the MS spectra and broken down using collision induced dissociation (CID) with atmospheric air as the collision gas, at a collision energy of 1kV and in the positive ion reflector mode. The precursor ions were selected within the mass windows of ±4Da. The non-redundant NCBI database (downloaded on 24/2/2011; 13135398 sequences) and in-house databases with the sorghum entries from NCBInr (71862 sequences) and the sorghum PEPC sequence only were used for protein identification, and the mass spectrometric data were searched using the MASCOT 2.3 server through the Global Protein Server v3.6 (AB Sciex). The search parameters were set up to no limitation in taxonomy, trypsin as the cleavage enzyme, carbamidomethylation on cysteine as the fixed modification, and the oxidation of methionine and ubiquitination (Gly-Gly adducts) of lysine, phosphorylation of serine or threonine as variable modifications. Mass tolerances of single MS and MS/MS were set up to 80ppm and 0.3Da, respectively. For trypsin digestion, one missed cleavage was allowed. In such analyses, the probability scores greater than the score fixed by mascot were considered to be significant at a *P*-value of <0.05. *De novo* sequencing of the MS/MS spectra of the peptides was performed using the DeNovo tool software (AB Sciex), the retrieved peptide sequences were manually checked and validated. A subsequent homology search of these sequences was obtained by BLAST (www.ncbi.nlm.nih.gov/BLAST).

PTPC PTMs were analyzed by a high-accuracy and sensitivity LC MS/MS. Coomassie blue-stained polypeptides were excised from SDS-PAGE gels, in-gel reduced, alkylated, and digested with trypsin using standard protocols (Uhrig *et al.*, 2008). Tryptic peptides were extracted using acetonitrile/0.1% trifluoroacetic acid (TFA) (v/v, 60:40), and dried using a CentriVap refrigerated centrifugal concentrator (Labconco Corp.). Peptides were reconstituted in 4 µl of 0.1% (v/v) formic acid (FA) and identified using two instrumentation methods: nanoAcquity ultra performance LC systems (Waters, Milford, MA) coupled with a TripleTOF 5600+ quadrupole time-of-flight mass spectrometer (AB Sciex, Concord, ON, Canada) at low-energy CID; and an Orbitrap Fusion Tribrid MS system (Thermo Fisher Scientific Inc.Watham, MA) in higher energy collision induced dissociation (HCD) mode. The sample was trapped by a 2G-V/MT Trap symmetry C18 column (5 μm particles, 180 μm id × 20mm length) at a flow rate of 5 μl min^–1^, and separated on a BEH130 C18 analytical column (1.7 μm particles, 100 µm id ×100mm length) at 300 nl min^−1^ for 60min. The mobile phase was set up to a linear gradient from 5–30% solvent B (0.1% FA in acetonitrile), followed by 85% solvent B over 10min for peptide elution. TripleTOF MS and MS/MS scans were acquired with a high resolution of 30.000, and the *m*/*z* regions of the MS survey scan and MS/MS were selected from *m*/*z* 350–1600 and *m*/*z* 100–1250, respectively, and by data-dependent scanning of the top twenty ions at multiply charged states of 2+, 3+ and 4+. Dynamic exclusion was set to a period of time of 30s. For comparison purposes, similar analyses were performed on the gel-separated proteins using the Orbitrap Fusion Tribrid LC-MS system under high-accuracy MS survey scan at a resolution of 60.000 followed by sequential HCD scans at the twenty top ions. MS/MS data from the two instruments were searched against the viridiplantae (green plants) protein sequences in the NCBI database using the Mascot Server (version 2.4.0, Matrix Science, London, UK). The search parameters were restricted to tryptic peptides at a maximum of two missed cleavages. Cysteine carbamidomethylation was designated as a fixed modification, and deamidation of asparagine and glutamine, oxidation of methionine, phosphorylation of serine/threonine/tyrosine, ubiquitination of lysine were considered as variable modifications. Mass tolerances were set up to 30ppm for TripleTOF 5600 MS ions and 0.05Da for MS/MS fragment ions, 20ppm for Orbitrap MS and 0.8Da for the MS/MS fragments. Peptide assignments were filtered by an ion score cut off at 15, and the identified MS/MS spectra were also verified manually.

### RNA Extraction and cDNA synthesis

Total RNA was isolated from 100mg of frozen, powdered half-embryonated germinating seeds or whole developing seeds using the IQeasy^TM^ Plant RNA Extraction (Intron Biotechnology). Extracted nucleic acids were DNase treated to eliminate genomic DNA. RNA was quantified using a NanoDrop 2000 spectrophotometer (Thermo Scientific). Reverse-transcription reactions were performed using 1 µg of purified total RNA, 1 µl ImProm-IITM Reverse Transcriptase (Promega) and a reaction buffer containing 0.5mM dNTP, 6mM MgCl_2_, 20 units recombinant RNasin® ribonuclease inhibitor, and 0.5 µg oligo(dt)15.

### qRT-PCR

Quantitative polymerase chain reaction (qRT-PCR) was performed in a final volume of 20 µl consisting of 1 µl of the cDNA, 10 µl of SensiFAST SYBR No-ROX kit (Roche) and 15 µM of the gene-specific primers pairs as follows: *SbPPC3* (forward 5′–TGTTGAACAGTTTCTGGAACCTCTT-3′, reverse 5′-GCTTCA CAAGGGCAAGCCCAAAG-3′), *SbPPC2* (forward 5′-CCGCCT CGCAACACCTGAAACA-3′, reverse 5′-ACCGGGAGGTGGAA CCGTGT-3′), *SbPPC4* (forward 5′-TGAGCTTCGGGCACAAGC AGATG-3′, reverse 5′-GCTCCAAAGGCTCTAAGAACTGCT C-3′), and *18 S* rRNA (forward 5′-GGGGAAACTTACCA GGTCCA-3′, reverse 5′-GGATGGCTCCGCATAGCTA-3′). PCR was conducted on the MiniOpticon^TM^ Real-Time PCR Detection System (Biorad), and the threshold cycles (Ct) were determined using Opticon Monitor^TM^ analysis software for all treatments. To normalize the obtained values, 18S ribosomal RNA was used as internal control in each sample. This gene displays a steady RNA level across the experimental conditions.

### Statistical analysis

Statistical analysis was performed using SigmaStat (Systat Software Inc., San José, CA, USA). Data were analyzed using the Student’s *t*-test or with the Mann-Whitney *U* test. Means were considered to be significantly different at *P*<0.05.

## Results and discussion

### PEPC activity, and PTPC integrity and cellular localization

**Fig. 1.**
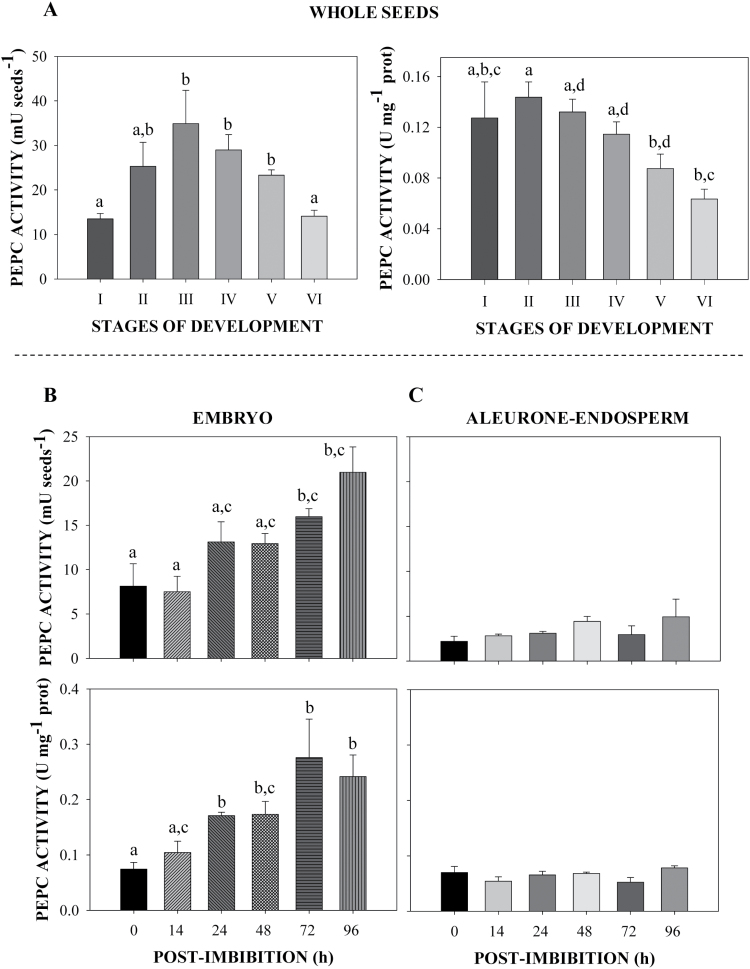
Time course of PEPC activity in developing and germinating sorghum seeds. PEPC activity was assayed at optimal pH (8.0), 2.5mM PEP, and 30 ºC in clarified extracts from: (A) whole seeds during development; and (B) embryos or (C) aleurone-endosperm of germinating seeds. (A) PEPC activity is expressed on a per seed basis (mU seeds^−1^) or on a protein basis (U mg^−1^ protein). Results are means ±SE of three independent experiments. Mean values that are not significantly different (*P*<0.05, Student’s *t*-test) are indicated with the same letters. Data for aleurone-endosperm are not significantly different.

PEPC was characterized throughout the development, maturation, and germination of sorghum seeds, focusing on the enzyme’s activity, integrity, subunit composition, and immunolocalization. Stages of sorghum seed development and germination are described in Supplementary Fig. S1 (at *JXB* online); among these, stage IV of development marks the beginning of seed desiccation, whilst stage II of germination marks the protrusion of the radicle and the completion of germination (Bewley *et al.*, 2013). During seed development, PEPC activity of desalted extracts increased by about 3-fold to reach a maximum level at stage II–III and then decreased (Fig. 1). During germination, PEPC activity in the embryo increased to a maximum at 72 to 96h post-imbibition, while in the aleurone/endosperm it did not change significantly and was 10–15-fold lower than that of embryos (Fig. 1).

**Fig. 2.**
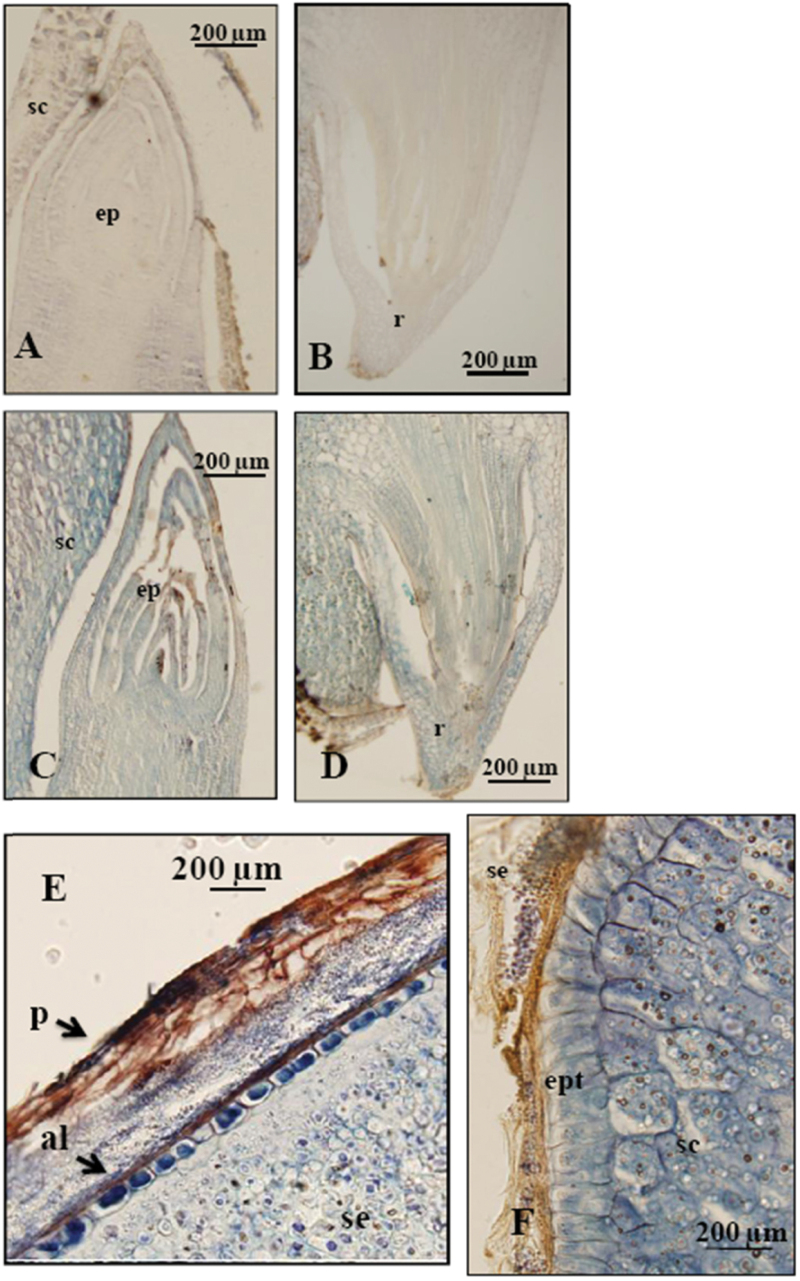
Immunological localization of PTPC in germinating sorghum seeds. Grains were imbibed for 24h. Sections (15 µm) were probed with: (A, B), preimmune serum, or (C–F) polyclonal anti-C_4_ PTPC (SbPPC1). Abbreviations: al, aleurone layer; ep, epicotyl; ept, epithelium; sc, scutellum; se, starchy endosperm; r, radicle; p, pericarp.

In germinating COS, the p110 and p107 PTPC polypeptides appear to be *in vivo* truncated by 19 amino acids such that their N-terminal phosphorylation domain is absent (Uhrig *et al.*, 2008). In contrast, non-monoubiquitinated p107 and monoubiquitinated p110 PTPCs from germinating sorghum seeds at 48h post-imbibition were shown to be intact and to therefore contain the phosphorylation motif (Ruiz-Ballesta *et al.*, 2014). In this study, we demonstrate the integrity of PTPC polypeptides throughout the life cycle of sorghum seeds since the anti-C19 and anti-N24 both cross-reacted with the p110 and p107 (see Supplementary Fig. S2). PTPC was also immunolocalized in germinating sorghum seed tissues characterized by high metabolic activities (epicotyl, radicle, aleurone layer, scutellum, scutellum’s epithelium) (Fig. 2). Immunohistochemistry studies have localized PTPC in the protein bodies of wheat grains, where it was suggested to contribute to amino acid and protein biosynthesis during grain development (Araus *et al.*, 1993). PTPC has also been localized in tissues of developing and germinating wheat grains characterized by high metabolic activity (González *et al.*, 1998). It is noteworthy that PEPC was detected as an abundant protein in the aleurone layer using immuno-localization (Fig. 2E). Consistent with this result, the role of PEPC in malate production by the barley aleurone layer has been described by Macnicol and Raymond, (1998).

Collectively, these results indicated the stability and consistent functional relevance of PTPC throughout the life cycle of sorghum seeds.

### Expression of PEPC genes in developing and germinating seeds

**Fig. 3.**
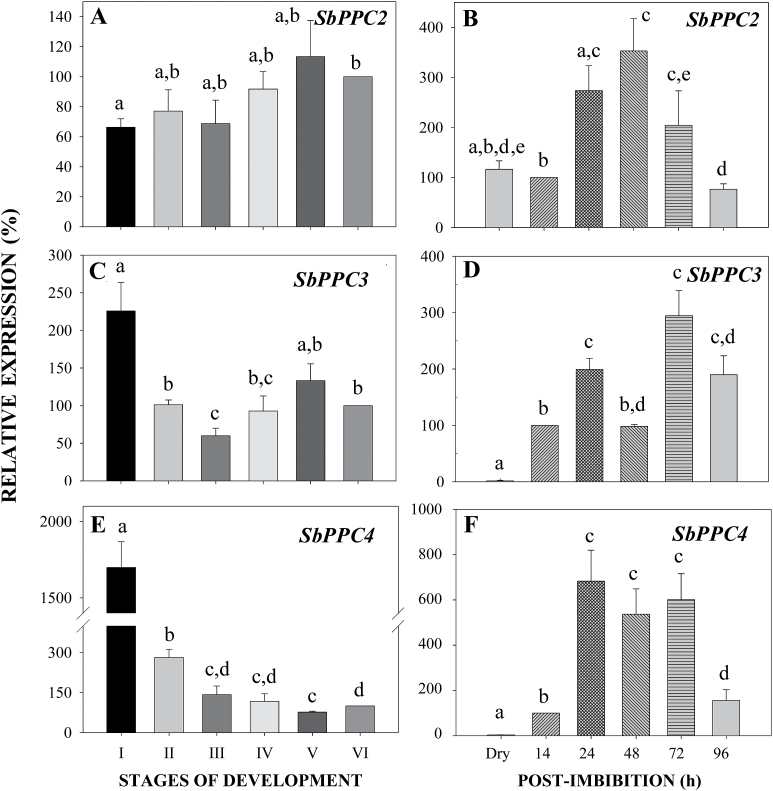
qRT-PCR analysis of *PTPC* transcript levels in developing and germinating sorghum seeds. To normalize the values, 18S RNA was used as internal control in each sample. Data were normalized to stage VI for developing seeds (A, C, E), or 14h post-imbibition germinating seeds (B, D, F). Results are means ±SE of at least three independent experiments. Mean values that are not significantly different (*P*<0.05, Student’s *t*-test) are indicated with identical letters.

Sorghum PEPC is encoded by a small multigene family consisting of six genes: *SbPPC2*, *SbPPC3*, *SbPPC4, SbPPC5* (all C_3_ PTPCs), and *SbPPC1* (C_4_ photosynthetic PTPC) encode closely related PTPCs, whilst *SbPPC6* encodes the distantly related BTPC (Table 1) (Paterson *et al.*, 2009). In this present study, we used qRT-PCR to analyze the pattern of their transcript abundance. *SbPPC2, SbPPC3*, and *SbPPC4* transcripts were present in developing and germinating sorghum seeds (Fig. 3). Transcripts of *SbPPC2*, the housekeeping PTPC (Lepiniec *et al.*, 1994), were detected during seed development and its abundance increased slightly during the maturation phase (Fig. 3A). In the germinating seeds, *SbPPC2* showed maximal transcript abundance at 24–48h post-imbibition and then decreased (Fig. 3B). By contrast, *SbPPC3* transcript levels were maximum at the beginning of seed development, during the period of cellularization (Bewley *et al.*, 2013); this was followed by a decrease of *SbPPC3* transcripts until stage III and then an increase during the desiccation period with a peak at stage V (Fig. 3C). *SbPPC3* transcripts were also detected during germination with two important peaks at 24 and 72h post-imbibition (Fig. 3D). Finally, *SbPPC4* transcripts were quite abundant at the beginning of development, suggesting a possible specific role for SbPPC4 during the phase of cellularization (Fig. 3E). Cellularization is the massive production of new cells. Malate is a ubiquitous molecule that provides carbon skeletons and reducing power and is needed for the synthesis of storage end-products by developing seeds. *SbPPC4* transcripts were also very abundant at 24–72h post-imbibition in germinating seeds (Fig. 3F). By contrast, transcripts of *SbPPC5* and *SbPPC6* (*BTPC*) were very low during sorghum seed development and germination and, as expected, no *SbPPC1* transcripts were detected (results not shown). These results show a novel pattern of the steady-state of transcript abundance of *PTPC* isogenes during the life cycle of sorghum seeds, with an important presence of *SbPPC3* and also an important contribution of the *SbPPC4* isogene to the cellularization stage of development (stage I) and during germination.

### Different PTPC isoenzymes co-exist in developing and germinating seeds

Peptide mass fingerprinting by MALDI-TOF MS of the p110 and p107 polypeptides immunoprecipitated using anti-C_4_ PTPC revealed that different PTPC isoenzymes co-exist in developing and germinating sorghum seeds (Table 2; asterisks denote confirmation of the identification via LC MS/MS sequencing of tryptic peptides). SbPPC3 was the most abundant during the course of the life cycle of the seeds in both p107 and p110. SbPPC3 was highly abundant in the embryo (Table 2) and less abundant in the aleurone/endosperm of the germinating seed; however, SbPPC3 was the only isoenzyme detected in this tissue (Table 2). The housekeeping SbPPC2 (Lepiniec *et al.*, 1993, 1994) was present in early development only as a p107 (i.e. probably non-monoubiquitinated) subunit (Table 2, stages I and III) and was identified as a p110 and p107 subunit in stage VI (Table 2). SbPPC2 was also present as a monoubiquitinated p110 subunit in the embryo of dry and 48-h post-imbibition seeds, but was not identified in the aleurone (Table 2). Finally, the *SbPPC4* isogene was shown to be translated into the corresponding p107 PTPC subunits in early development (Table 2, stages I and III), in agreement with the transcript profiling results of Fig. 3E. In addition, SbPPC4 was also detected in p110, indicating the probable monoubiquitination of this PTPC isoenzyme. Although the precise physiological function of SbPPC4 remains unclear, our results support a role for this isoenzyme during the early stages of seed development. During the phase of cellularization SbPPC4 is hypothesized to generate the C-skeletons needed as precursors for synthesis of new components such as amino acids/proteins, and membrane or storage lipids. Finally, SbPPC5 was detected only as p110 polypeptides in stage I (Table 2). SbPPC6 polypeptides were never detected by MS/MS analysis, in agreement with the absence of *SbPPC6* expression during sorghum seed development and germination. Nor was a high molecular mass Class-2 PEPC hetero-octamer evident when clarified extracts from different stages of development and germination were subjected to non-denaturing PAGE followed by in-gel PEPC activity staining (see Supplementary Fig. S3). However, tetrameric and dimeric Class-1 PEPC oligomers were observed, with the dimeric form being the most abundant *in vitro* (Supplementary Fig. S3A). This dimeric form was not altered by de-ubiquitination of the enzyme after incubation with 2 µM of the catalytic subunit of ubiquitin specific protease-2 (USP-_2c_) (Supplementary Fig. S3B).

**Table 2.** Identification of p107 and p110 PTPC isoforms from developing and germinating sorghum seeds. Proteins were identified via MALDI-TOF/TOF MS peptide mass fingerprinting of tryptic peptides derived from immunoprecipitated samples of representative stages. Asterisks indicate identification via MS/MS sequencing of tryptic peptides and BLAST analysis. DPA, days post-anthesis.

**STAGE**	**Subunit**	**Protein name**	**Accession number** ^**a**^	**Score**	**Sequence coverage (%**)	**N** ^**o**^ **. of Matching Peptides**
**I** (7–12 DPA)	**p110**	PEPC SbPPC3*	gi|241931686	248	35	31
		PEPC SbPPC4*	gi|241940554	118	24	22
		PEPC SbPPC5	gi|241930496	98	22	21
	**p107**	PEPC SbPPC3	gi|241931686	325	45	39
		PEPC SbPPC4	gi|241940554	114	26	21
		PEPC SbPPC2	gi|241925556	107	23	20
**III** (16–20 DPA)	**p110**	PEPC SbPPC3	gi|241931686	103	24	21
	**p107**	PEPC SbPPC3	gi|241931686	206	33	29
		PEPC SbPPC4	gi|241940554	66	19	16
		PEPC SbPPC2	gi|241925556	61	17	15
**VI** (31–40 DPA)	**p110**	PEPC SbPPC3	gi|241931686	60	10	13
		PEPC SbPPC2*	gi|241925556	36	9	8
	**p107**	PEPC SbPPC3	gi|241931686	121	15	18
		PEPC SbPPC2*	gi|241925556	76	8	10
**Dry seed** Aleurone/endosperm	**p110**	PEPC SbPPC3	gi|241931686	30	13	9
	**p107**	PEPC SbPPC3	gi|241931686	72	20	15
**Dry seed** Embryo	**p110**	PEPC SbPPC3*	gi|241931686	310	45	39
		PEPC SbPPC2	gi|241925556	91	21	18
	**p107**	PEPCSb PPC3	gi|241931686	283	42	48
		PEPC SbPPC2	gi|241925556	63	16	14
**48h post-imbibition** Aleurone/endosperm	**p110**	PEPC SbPPC3*	gi|241931686	95	24	16
	**p107**	PEPC SbPPC3	gi|241931686	67	16	15
**48h post-imbibition** Embryo	**p110**	PEPC SbPPC3	gi|241931686	305	44	38
		PEPC SbPPC2	gi|241925556	54	15	14
	**p107**	PEPC SbPPC3*	gi|241931686	164	29	38
		PEPC SbPPC2	gi|241925556	55	15	13

^a^NCBI database

A number of important points emerge from these results, as follows. (i) Multiple C_3_ PTPC isoenzymes are present during the development and the germination of sorghum seeds. SbPPC3 appears to have an essential role as it is the isoenzyme most consistently present during the course of the life cycle of the seeds , and was the only one detected in the aleurone/endosperm. (ii) The SbPPC4 isozyme appears to play a major role during the very early stage of seed development, known as the phase of cellularization (Bewley *et al.*, 2013). (iii) BTPC is not significantly expressed throughout the life cycle of the seeds, which is an important difference relative to developing COS, a non-green oil seed, in which p118 BTPC polypeptides, and thus Class-2 PEPC complexes are highly expressed (O’Leary *et al.*, 2011*b*).

### Post-translational modifications of the different Class-1 PEPC isoenzymes during germination

In the endosperm of germinating COS, p110 PTPC subunits are monoubiquitinated but not phosphorylated (Uhrig *et al.*, 2008; O’Leary *et al.*, 2011*b*). On the other hand, simultaneous p110 monoubiquitination and p107 phosphorylation have been described as an alternative PTM pattern for Class-1 PEPC of immature proteoid (cluster) roots of harsh hakea (Shane *et al.*, 2013). However, the coincident occurrence of both PTMs in sorghum seed p110 and p107 PTPC (i.e. SbPPC3) subunits has only been shown *in vitro* (Ruiz-Ballesta *et al.*, 2014). In this study, we show both PTMs also occur *in vivo* with three of the four PTPC isoenzymes present in germinating sorghum seeds. To this end, PTPC polypeptides of clarified extracts from 48-h post-imbibition germinating seeds were isolated by immunoaffinity chromatography with anti-COS PEPC (see Supplementary Fig. S4A, B). Coomassie blue R-250-stained p110 and p107 were excised from SDS gels of the immunopurified PTPC and individually subjected to in-gel tryptic digestion and detailed analysis by LC MS/MS. A Mascot database search of p110 and p107 datasets derived from nano HPLC TripleTOF MS of their tryptic peptides (Table 3) revealed the presence of SbPPC2, SbPPC3, and SbPPC4 PTPCs in p110 and p107, as well as SbPPC5 in p107. Consistent with our earlier results (Ruiz-Ballesta *et al.*, 2014), p107 N-terminal tryptic peptides of SbPPC3 containing non-phosphorylated Ser7 were identified by Orbitrap LC MS/MS. However, the monoubiquitinated SbPPC3 p110 subunits were also determined to be phosphorylated at Ser7 (Table 3). Monoubiquitinated SbPPC2 and SbPPC4 (i.e. p110 subunits), and deubiquitinated SbPPC5 (i.e. p107 subunits) were also shown to be phosphorylated at their conserved N-terminal seryl phosphorylation sites; i.e. Ser13, Ser10, and Ser11 for SbPPC2, SbPPC4, and SbPPC5, respectively (Table 3). To the best of our knowledge, this is the first evidence that *in vivo* phosphorylation of different PTPC isoenzymes can simultaneously occur in any plant tissue. MS/MS also confirmed the monoubiquitination of SbPPC3 p110 subunits at Lys624 (Table 3), as previously reported (Ruiz-Ballesta *et al.*, 2014). In addition, TripleTOF MS/MS analysis of the quadruply charged SbPPC2 p110 peptide ion of *m*/*z* 455.7462 (LC retention time 16.61min) and the triply charged ion of *m*/*z* 507.9268 (LC retention time 18.01min), corresponding to residues 623–637 (VAKDFGVKLTMFHGR) and 626–637 (DFGVKLTMFHGR), showed that the masses of the C-terminal fragments up to y7 matched the predicted values, but the mass shift of the fragment y8 by 114Da indicated a ubiquitination site localized at Lys-630, where a characteristic mass increment of 114Da indicates a Gly-Gly motif attachment of ubiquitin at the side chain (Supplementary Fig. S5A, B). LC MS/MS measurements of the doubly and triply charged ions of *m*/*z* 769.3827 and *m*/*z* 513.2607 (LC retention time 23.22min) using a high-resolution Orbitrap Fusion Tribrid instrument identified the ubiquitination of the oxidized peptide at residues 626–637 (Supplementary Fig. S5C, D). Under high-resolution HCD fragmentation, the mass shift of 114Da on both the N-terminal *b5* ion and the C-terminal *y8* ion unambiguously confirmed Lys-630 as p110’s monoubiquitination site in SbPPC2. This residue precisely aligns with the Lys-624 and Lys-628 (a highly conserved PTPC residue that is immediately proximal to catalytic residues involved in PEP binding) monoubiquitination sites, respectively determined for p110 subunits of sorghum seed SbPPC3 PTPC (Table 3 and Ruiz-Ballesta *et al.*, 2014) and PTPC (RcPPC3) from germinated COS (Uhrig *et al.*, 2008).

**Table 3.** Identification of PTMs of immunopurified PTPC polypeptides from 48h germinated sorghum seeds using TripleTOF 5600 and Orbitrap Fusion mass spectrometry. pSer; phosphorylated-Ser; Ub, monoubiquitinated.

**Peptide**	**m/z(obs.**)	**Mr(exp.**)	**Mr(calc.**)	**ppm**	**Score**	**Peptide sequence**	**Modification sites**
**p107:** PEPC SbPPC2, 107kDa, NCBI access # gi|242048870, sequence coverage 90%.							
8–19	749.8647	1497.7148	1497.7010	9.2	18	K.MERLS***S***IDAQLR.M	pSer13
11–19	541.7654	1081.5163	1081.5169	-0.5	49	R.LS***S***IDAQLR.M	pSer13
**p107:** PEPC SbPPC3, 107kDa, NCBI access # gi|242061132, sequence coverage 87%.							Not detected
**p107**: PEPC SbPPC4, 107kDa, NCBI access # gi|242078871, sequence coverage 89%.							
8–16	542.7460	1083.4764	1083.4784	-1.9	34	K.MA***S***IDAQLR.M	pSer10
8–16	550.7449	1099.4753	1099.4733	1.8	34	K.MA***S***IDAQLR.M	pSer10; Met-Ox
**p107**: PEPC SbPPC5, 107kDa, NCBI access # gi|242058751, sequence coverage 64%.							
4–17	527.9304	1580.7694	1580.7559	8.5	16	R.NAVDKAT***S***IDAQLR.L	pSer11
4–17	791.3842	1580.7528	1580.7559	2.0	58	R.NAVDKAT***S***IDAQLR.L	pSer11
**p110:** PEPC SbPPC2, 110kDa, NCBI access # gi|242048870, sequence coverage 94%.							
8–19	749.8642	1497.7128	1497.7010	8.2	22	K.MERLS***S***IDAQLR.M	pSer13
11–19	541.7646	1081.5146	1081.5169	-2.1	43	R.LS***S***IDAQLR.M	pSer13
623–637	455.7462	1818.9535	1818.9563	1.6	110	K.VAKDFGV***K***LTMFHGR.G	UbLys-630
626–637	507.9268	1520.7570	1520.7558	-1.0	60	K.DFGV***K***LTMFHGR.G	UbLys-630
626–637	513.2607	1536.7587	1536.7507	5.5	19	K.DFGV***K***LTMFHGR.G	UbLys-630, Met-OX
626–637	769.3827	1536.7498	1536.7507	1.0	30	K.DFGV***K***LTMFHGR.G	UbLys-630, Met-OX
**p110:** PEPC SbPPC3, 110kDa, NCBI access # gi|242061132, sequence coverage 93%.							
2–13	765.3659	1528.7173	1528.7147	1.6	19	M.PERHQ***S***IDAQLR.L	pSer7
620–631	780.4022	1558.7888	1558.7827	4.1	90	K.HYGV***K***LTMFHGR.G	UbLys-624
620–631	390.7011	1558.7752	1558.7827	-4.8	42	K.HYGV***K***LTMFHGR.G	UbLys-624
620–631	520.5975	1558.7707	1558.7827	-7.6	71	K.HYGV***K***LTMFHGR.G	UbLys-624
**p110**: PEPC SbPPC4, 110kDa, NCBI access # gi|242078871, sequence coverage 90%.							
8–16	542.7472	1083.4798	1083.4784	1.3	17	K.MA***S***IDAQLR.M	pSer10
8–16	550.7423	1099.4700	1099.4733	-3.0	37	K.MA***S***IDAQLR.M	pSer10

Collectively, these results establish for the first time several important points. (i) Different PTPC isoenzymes are co-expressed during sorghum seed germination and all of them except the p107 subunits of SbPPC3 are phosphorylated during germination. (ii) Monoubiquitination of the corresponding conserved Lys was confirmed by MS/MS analysis for SbPPC3, and determined in this study for SbPPC2, demonstrating that this PTM occurs with multiple PTPC isoenzymes at a conserved lysine residue within a single plant species. (iii) Monoubiquitination and phosphorylation can simultaneously occur on the same PTPC polypeptide and are therefore not mutually exclusive PTMs, as appears to be the case with PTPCs from developing proteoid roots of harsh hakea (Shane *et al.*, 2013), as well as various green and non-green tissues of the castor oil plant (Uhrig *et al.*, 2008; O’Leary *et al.*, 2011*b*).

### Is PTPC monoubiquitination linked to specific stages and/or environmental conditions during sorghum seed germination?

Because of its recent discovery (Uhrig *et al.*, 2008), there are few studies about the influence of environmental factors or biotic/abiotic stresses on PTPC monoubiquitination during seed development and germination. Elimination of photosynthate translocation (a type of abiotic stress) from the shoot to the seed by depodding (fruit excision) of developing COS triggered rapid *in vivo* p107 dephosphorylation, followed by monoubiquitination of 50% of the p107 subunits to form p110 characteristic of germinating COS (O’Leary *et al.*, 2011b).

**Fig. 4.**
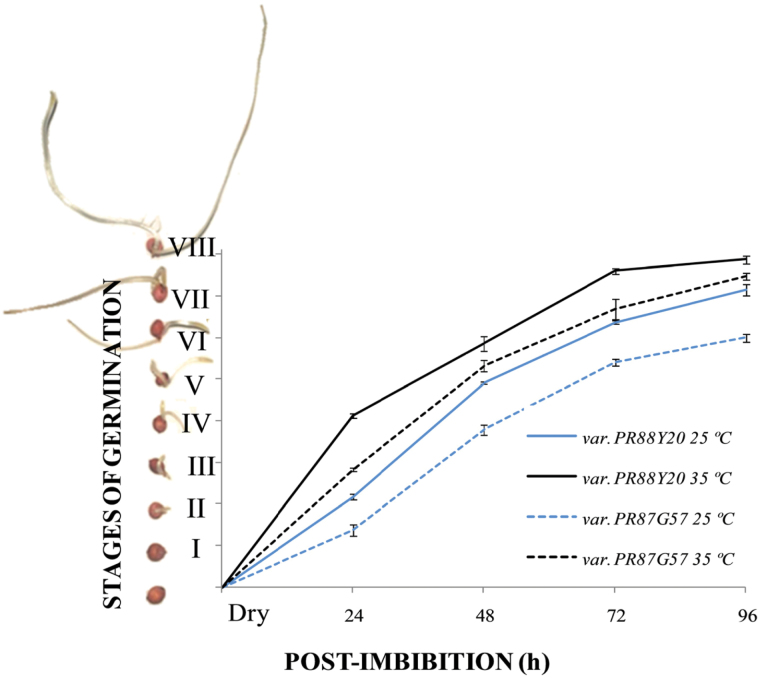
Germination rate of two different sorghum varieties at 25 and 35 ºC. PR88Y20 and PR87G57 were germinated from dry seed to 96h post-imbibition. The average stage of germination was calculated as described in the Materials and Methods. Results are means ±SE of at least three independent experiments.

**Fig. 5.**
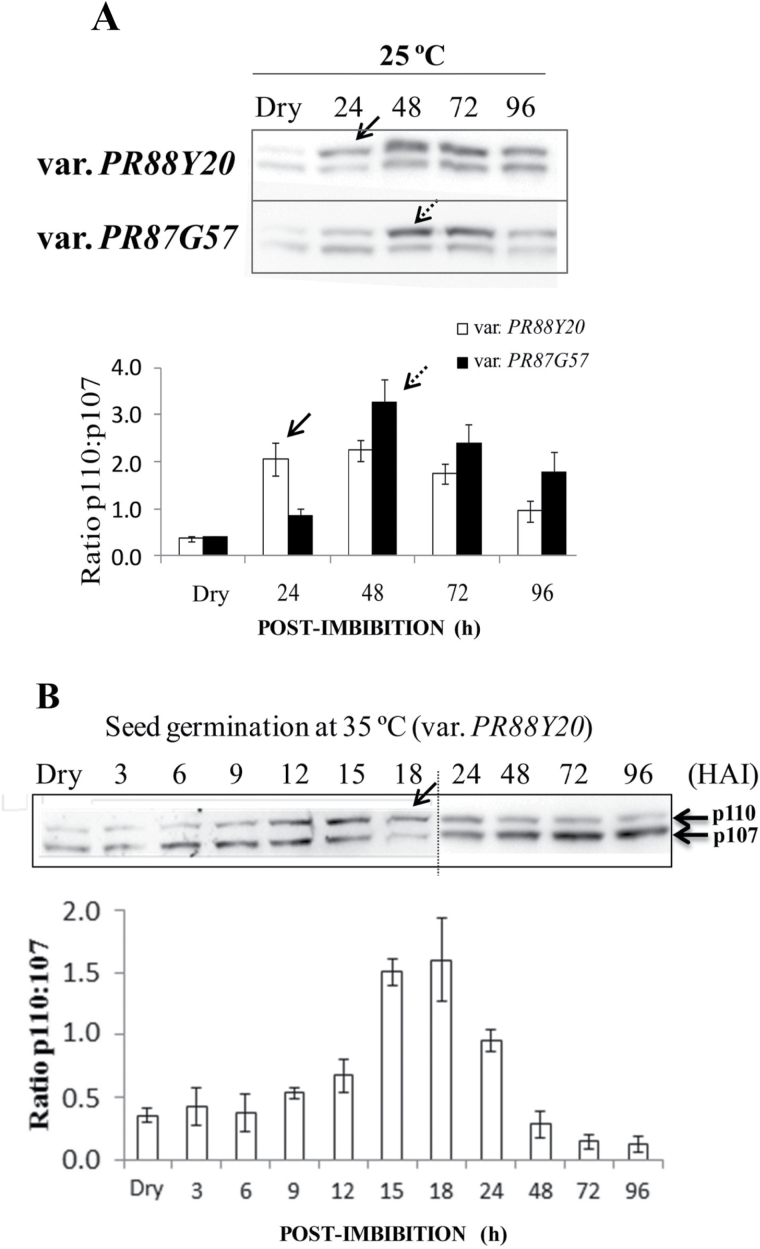
Patterns of PTPC monoubiquitination in two different varieties of sorghum at 25 and 35 ºC. (A) Clarified extracts from dry seeds to 96h post-imbibition were subjected to 8% SDS-PAGE (50 µg protein lane^−1^) and analyzed by immunoblotting using anti-C_4_ PTPC SbPPC1. The plot represents the ratio of p110:p107 determined from scanned immunoblots. (A) and (B) show seeds imbibed at 25 or 35 ºC, respectively. Results are means ±SE of at least three independent experiments. The arrows indicate the maximum level of monoubiquitination and p110:p107 ratio. HAI, hours after imbibition.

Sorghum is a cereal cultivated in warm environments and seeds germinate well within a wide range of temperature from 25–35 ºC, with an optimum of over 30 ºC (Kanemasu *et al.*, 1975). This allowed us to correlate the effect of temperature with germination and the pattern of PTPC monoubiquitination (i.e. appearance of p110 polypeptides on PTPC immunoblots). To this end, seeds from two sorghum varieties PR88Y20 and PR87G57, respectively characterized by rapid and slow germination velocities, were imbibed in water for up to 96h at 25 and 35 ºC. As defined by Bewley *et al.* (2013), germination begins with water uptake by the seed (imbibition, Phase I) and ends with the emergence of the radicle, which marks the end of Phase II and the beginning of Phase III (Bewley *et al.*, 2013). Phase II is also associated with a slow rate of water uptake, where the little net movement of water into the seed is due mainly to a lowering of the solute potencial (*ψ*_s_), which drives an increase in volume that results in cracking of the testa. Germination does not include seedling growth. Accordingly, eight stages of germination were established in this study (see Supplementary Fig. S1). Among them stage II represents the emergence of the root, and the beginning of the post-germination Phase III (Bewley *et al.*, 2013). Germination rates were calculated by determining the average of the different stages of germination in a pooled of seeds at a given time post-imbibition (0, 24, 48, 72, and 96h). Figure 4 shows germination rate for the two sorghum varieties at 25 and 35 ºC. Both varieties had a higher germination velocity at 35 than 25 ºC, with the seeds germinating at 35 ºC having an average lead of two stages over those incubated at 25 ºC (Fig. 4). Moreover, PR88Y20 germinated more rapidly than PR87G57 at both 25 and 35 ºC (Fig. 4). In parallel, clarified extracts from the same pool of seeds (the 15–20 seeds in the most advanced stages of germination at each time of imbibition) were analyzed by SDS-PAGE and immunoblotting with anti-C_4_ PEPC (SbPPC1) (Fig. 5). PR88Y20 at 25 ºC attained maximal PTPC monoubiquitination (i.e. formation of p110) between 24 and 48h post-imbibition (Fig. 5A, arrow), with a p110:p107 ratio of approximately 2. By contrast, maximal PTPC monoubiquitination in PR87G57 appeared to be delayed by up to 48–72h, in accordance with its slower germination rate (Fig. 5A, dashed arrow), with a p110:p107 ratio of 3 at 48h. PTPC monoubiquitination in seeds germinated at 35 ºC occurred much earlier (Fig. 5B, var. PR88Y20), starting between 9 and 12h post-imbibition and reaching a maximal p110:p107 ratio (around 1.7) at 18h (Fig. 5B, arrow). These results indicate a strong correlation between the germination and post-germination velocity and the pattern of monoubiquitination of p110 PTPC subunits. Further analysis of the results indicated that, regardless of the time post-imbibition, PTPC monoubiquitination starts around stage II of germination for each variety or temperature and decreases around stage IV–V (Fig. 4). Thus PTPC monoubiquitination is well correlated with Stage II’s period of high cell division during which there is massive mobilization of reserves, and corresponding biosynthesis of the new macromolecules needed for development of the growing seedling. In addition, PTPC monoubiquitination decreased around stage IV–V when the coleoptile is about 1cm long and becomes green to initiate photoautotrophic seedling growth.

**Fig. 6.**
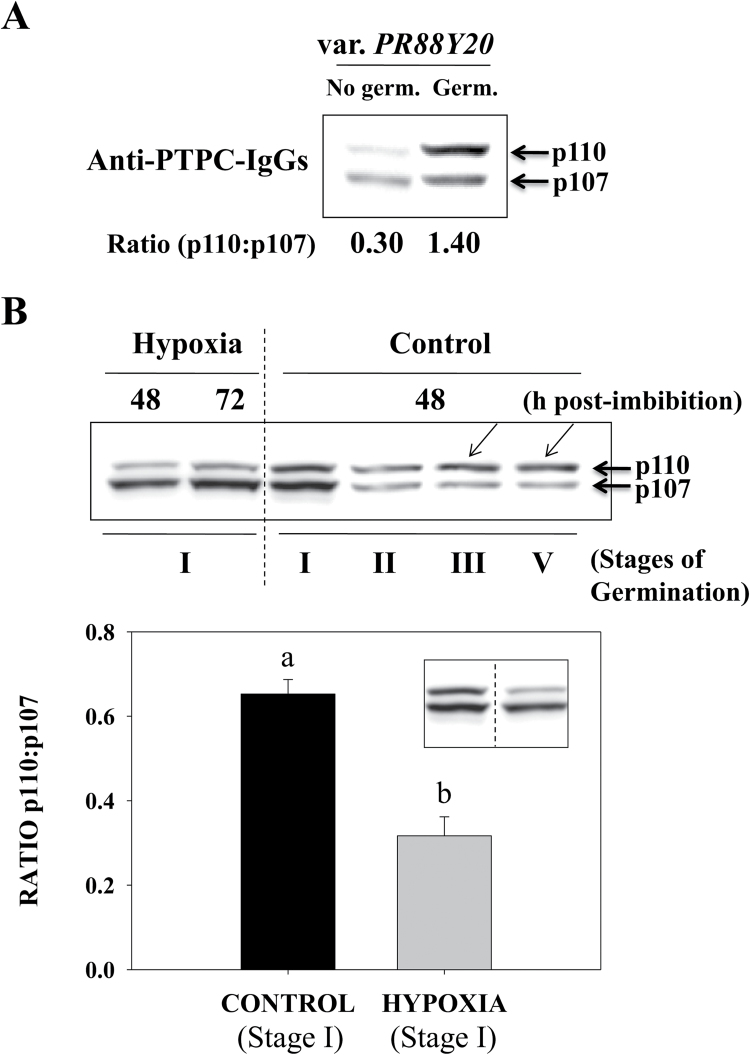
PTPC monoubiquitination occurs in viable seeds that have increased aerobic respiration and reserve mobilization associated with radicle emergence. (A) Seeds (var. PR88Y20) at 24h post-imbibition were separated into two pools: germinated seeds (where the testa was cracked with protrusion of the radicle; Germ) and non-germinated seeds (where the testa was not disrupted, No Germ.). Clarified extracts were subjected to 8% SDS-PAGE and analyzed by immunoblotting with anti-C_4_ PTPC. (B) Seeds were imbibed submerged in water (Hypoxia) or on moistened filter paper (Control, normoxic). At 48h post-imbibition control seeds mostly ranged from stage II to IV of germination, although some remained at stage I (Control, stages I, II, III and IV), while hypoxic seeds remained at stage I even at 72h post-imbibition. Results in the plot represent the ratio of p110:p107 in control and hypoxic seeds at stage I, 48h post-imbibition, and represent means ±SE of at least three independent experiments. Mean values that are significantly different (*P*<0.01, Student’s *t*-test) are indicated with different letters.

To further analyze this phenomenon, seeds were imbibed with water for periods up to 24h and classified as germinated seeds (stage II) or non-germinated seeds (stage I) depending on the emergence or not of the radicle. PTPC monoubiquitination was far more prevalent in germinating seeds where the emergence of the radicle occurred (Fig. 6A, Germ.). These results confirmed that PTPC monoubiquitination in germinating sorghum seeds coincides with metabolic events associated with the emergence of the radicle (stage II) and early seedling growth.

As the relative rates of glycolysis and gluconeogenesis and adenylate energy charge are influenced by oxygen availability (Bewley *et al.*, 2013), it was also of interest to study the impact of hypoxia stress on PTPC monoubiquitination during seed germination. To this end, seeds were imbibed on moist filter paper (control, normoxic) or submerged in water (hypoxic). At 48h post-imbibition most of the normoxic seeds reached stage II, a smaller number remained at stage I, whereas others had attained stage III–IV (Fig. 6, control). However, the submerged seeds remained at stage I, at 72h post-imbibition (Fig. 6, Hypoxia). The hypoxia-stressed seeds were not dead since they germinated well after they were transferred to normoxic conditions (data not shown). As expected, PTPC monoubiquitination of normoxic seeds markedly increased at stages II to III, but remained low in hypoxia-stressed seeds, even up to 72h post-imbibition (Fig. 6B). These results establish that enhanced PTPC monoubiquitination is intrinsically linked to the germination of the seed and corresponding enhanced aerobic respiration, cracking of the testa, and the protrusion of the radicle. This makes sense since the ubiquitination machinery is ATP dependent, and abundant ATP probably requires abundant aerobic respiration (Callis, 2014). In addition, even if germination did not occur (i.e. the radicle did not emerge; stage I), PTPC monoubiquitination was enhanced under normoxic relative to hypoxic conditions (Fig. 6B).

These are the first results showing that PTPC monoubiquitination in germinating sorghum seeds: (i) is very low during Phase I; (ii) always increases at stage II (emergence of the radicle); (iii) is maintained during the aerobic period of high activity of cell division and reserve mobilization; and (iv) remains until stage IV–V when coleoptiles are about 1cm long and the formation of the photosynthetic tissues begins.

## Concluding remarks

Deciphering the occurrence, roles, and PTMs of different PTPC isoenzymes during seed development and germination has been poorly studied. Monoubiquitination was recently discovered to be a novel but relatively common PTM of PTPC subunits of Class-1 PEPCs from diverse tissues and plant species (Uhrig *et al.*, 2008; Igawa *et al.*, 2010; O’Leary *et al.*, 2011b; Ruiz-Ballesta *et al.*, 2014; Shane *et al.*, 2013). However, the occurrence of PTPC monoubiquitination and phosphorylation has been specifically described only in the limited examples of developing and germinating COS (Tripodi *et al*., 2006; Uhrig *et al.*, 2008; O’Leary *et al.*, 2011*b*), and during proteoid root formation in harsh hakea (Shane *et al.*, 2013). This present study describes the patterns of transcript levels and relative abundance of different PTPC isozymes during sorghum seed development and germination, as well as their PTM by monoubiquitination and phosphorylation during germination. The results reveal new and important points; for example, the high level of *SbPPC4* transcripts during early development, at stage I (Fig. 3). This is the first report in which a possible role of this PTPC isozyme during cellularization of the developing seed (Bewley *et al.*, 2013) is suggested. The absence of *BTPC* transcripts and protein represent an important difference with developing COS, a non-green oil seed in which BTPC is highly expressed and appears to play an important role as a catalytic and regulatory subunit of the unusual Class-2 PEPC hetero-octameric complex (Uhrig *et al.*, 2008; O’Leary *et al.*, 2011*a*, *b*). *SbPPC2* and *SbPPC3* transcripts and SbPPC2 and SbPPC3 isoenzymes were extensively detected throughout the life cycle of sorghum seeds (Fig. 3 and Table 2). As SbPPC3 expression in roots depends on nitrogen supply (Lepiniec *et al.*, 1993), we propose an important role of this enzyme in providing the precursors needed for amino acid synthesis and transamination reactions in the germinating seed. In addition, SbPPC3 was the only isoenzyme found in the aleurone/endosperm, where it is proposed to play an important role in regulating the production of malic acid, which is either excreted for the acidification of the starchy endosperm (Macnicol and Raymond, 1998), or transported to the growing seedling for the anaplerotic function (Osuna *et al.*, 1996, 1999). Among the immunopurified PTPCs revealed by MS/MS analysis SbPPC2, SbPPC3, and SbPPC4 are monoubiquitinated *in vivo*. These results provide the first evidence that monoubiquitination is not restricted to a single PTPC isoenzyme within a specific plant species or physiological context, but can occur with multiple PTPC isozymes *in vivo*. SbPPC2, SbPPC3, and SbPPC4 were simultaneously monoubiquitinated and phosphorylated (Table 3), showing that both PTMs can occur *in vivo* in the same PTPC polypeptide (Table 3); i.e., they are not mutually exclusive PTMs, as appears to be the case in castor oil plants (Uhrig *et al.*, 2008; O’Leary *et al.*, 2011*b*). Class-1 PEPC phosphorylation at the single highly conserved serine residue close to the N-terminus of the protein uniformly results in the enzyme activation at physiological pH by decreasing and increasing its sensitivity to allosteric inhibitors and activators, respectively (Echevarría *et al.*, 1994). By contrast, PTPC monoubiquitination has an opposite kinetic effect (Uhrig *et al.*, 2008; Shane *et al.*, 2013). In germinating COS and developing proteoid roots of P-deficient harsh hakea, both PTPC PTMs appear to be exclusive, leading to a clear picture about the impact of each PTM on the activity of the enzyme. However, we show in this study that monoubiquitination and phosphorylation can simultaneously occur with three of the four PTPC isoenzymes expressed in sorghum seeds. This suggests that a combination of both PTMs may serve to fine tune the precise kinetic and regulatory properties for each PTPC isoenzyme at each specific stage of development and germination. However, it is at present difficult to establish the precise impact of phosphorylation versus monoubiquitination in each PTPC isoenzyme at each stage of the life cycle of sorghum seed. The use of phosphorylation-site-specific PTPC antibodies (i.e. anti-pSer13 and anti-pSer7 for SbPPC2 and SbPPC3, respectively), which we are presently characterizing, will help us to evaluate the specific pattern of *in vivo* phosphorylation for the two predominant PTPC isoenzymes in sorghum seeds. In this study we have documented the remarkable versatility and complexity of *in vivo* PTMs of different PTPC isoenzymes during the life cycle of sorghum seeds.

This is also the first study where the extent of PEPC monoubiquitination was observed to increase at stage II of germination. This stage corresponds to radicle protrusion and the beginning of the post-germination phase (Phases II and III, respectively, as described by Bewley *et al.*, 2013), and also to initiation of the second respiratory burst due to the cracking of the testa (Bewley *et al.*, 2013).

Future studies are needed in order to clarify the extent of PEPC monoubiquitination in other seeds, its relation with the efficiency of germination, and its possible regulation by metabolites and other elements of the signal transduction pathway that lead to this PTM. It will also be important to assess whether this PTM influences PEPC’s stability, protein–protein interactions, and/or subcellular location. It will be equally important to identify how this PTM is co-ordinated with PPCK-mediated phosphorylation-activation of PTPCs at their conserved N-terminal seryl residue. We are currently attempting to disrupt *PPCK* and *SbPPC3* expression in transgenic sorghum in order to study the impact of the absence of PTPC phosphorylation (*PPCK* silenced), or the SbPPC3 PTPC isoenzyme on seed development and germination.

## Supplementary data

Supplementary data are available at *JXB* online.

Figure S1. Stages of development and germination in sorghum seeds.

Figure S2. Pattern of PTPC subunit structure and integrity during sorghum seed development and germination.

Figure S3. Non-denaturing PAGE followed by in-gel PEPC activity staining of sorghum seed extracts.

Figure S4. Immunopurification of PTPC (p110 and p107) from germinating sorghum seeds

Figure S5. The p110 subunit of the SbPPC2 isoenzyme immunopurified from germinating sorghum seeds (48h post-imbibition) is monoubiquitinated at Lys-630.

Supplementary Data

## Abbreviations:

BTPC: bacterial-type phosphoenolpyruvate carboxylase
COS: castor oil seed
DTT: dithiothreitol
Glc-6-P: glucose-6-phosphate
MALDI TOF MS: matrix-assisted laser desorption ionization time-of-flight mass spectrometry
p110 and p107: 110 and 107-kDa polypeptides, respectively
PEP: phosphoenolpyruvate
PEPC: PEP carboxylase
PPCK: PEPC-kinase
PTPC: plant-type phosphoenolpyruvate carboxylase
PTM: post-translational modification.
