# Supplementary material for: New insights into the post-translational modification of multiple phosphoenolpyruvate carboxylase isoenzymes by phosphorylation and monoubiquitination during sorghum seed development and germination
Source: J Exp Bot. 2016 May 18;67(11):3523–36. doi: 10.1093/jxb/erw186 (PMC4892742; doi:10.1093/jxb/erw186)
Supplement: Supplementary Data [file supp_67_11_3523__index.html]

New insights into the post-translational modification of multiple phosphoenolpyruvate carboxylase isoenzymes by phosphorylation and monoubiquitination during sorghum seed development and germination — New insights into the post-translational modification of multiple phosphoenolpyruvate carboxylase isoenzymes by phosphorylation and monoubiquitination during sorghum seed development and germination — Supplementary Data 

# New insights into the post-translational modification of multiple phosphoenolpyruvate carboxylase isoenzymes by phosphorylation and monoubiquitination during sorghum seed development and germination

## Supplementary Data

Data files

- Supplementary\_figures\_S1\_S5.pdf - Supplementary Data
