# Supplementary material for: New insights into the post-translational modification of multiple phosphoenolpyruvate carboxylase isoenzymes by phosphorylation and monoubiquitination during sorghum seed development and germination
Source: J Exp Bot. 2016 May 18;67(11):3523–36. doi: 10.1093/jxb/erw186 (PMC4892742; doi:10.1093/jxb/erw186)
Supplement: Supplementary Data [file supp_erw186_Supplementary_figures_S1_S5.pdf]

Journal of Experimental Botany

New insights into the post-translational modification of multiple phosphoenolpyruvate carboxylase isoenzymes by phosphorylation and monoubiquitination during sorghum seed development and germination.

Isabel Ruiz-Ballesta[1], Guillermo Baena[1], Jacinto Gandullo [1], LiqunWang [2,3], Yi-Min She[2], William Charles Plaxton[4] and Cristina Echevarría[1,]*

Supplementary Materials and Methods

Antibodies

Anti-C19 and anti-N24 were respectively raised against synthetic peptides corresponding to the C-terminal [(Y) EDTLILTMKGIAAGMQNTG] and dephosphorylated N-terminal [ERHHSIDAQLRALAPGKVSEE24(YG)] ends of SbPPC5 (*i.e.,* sorghum C_4-photosynthetic PTPC) as previously described (Ruiz-Ballesta *et al.,* 2014). Anti-COS PTPC was raised against native Class-1 PEPC purified from endosperm of developing castor oil seeds as described in Tripodi *et al*. (2005).

Supplementary Figure Legends

Supplementary Figure S1. Stages of sorghum seed development and germination in . Seeds were harvested at different days post-anthesis (DPA), frozen in liquid N_2, and stored at -80 ºC until used. Mature sorghum seeds were sterilized and imbibed on filter paper moistened with sterile water in a glass petri dish. Seeds were germinated for up to 96 h at room temperature (25 ºC) and separated into different stages (from I to VIII). *Phases (P) of germination described by Bewley *et al*., (2013). PI, imbibition; PII, germination *sensu stricto.* PIII, post-germination.

Supplementary Figure S2. Pattern of PTPC subunit structure and integrity during sorghum seed development and germination. At the indicated stages of seed development (A-C) or germination (D-G), soluble proteins (80 µg/lane) of clarified extracts were resolved by SDS-PAGE (8% acrylamide), transferred onto nitrocellulose, and probed with different PTPC antibodies as described in the

Supplementary Materials and methods and indicated in the respective panels. Panels A, B, C, E and F are representative immunoblots of whole seed extracts, whereas panels D and G are immunoblots of embryo and aleurone extracts, respectively. Immunoreactive polypeptides were visualized using a chemiluminescence detection system (Super Signal West Dura Signal; Pierce) according to the manufacturer's instructions.

Supplementary Figure S3. Non-denaturing PAGE followed by in-gel PEPC activity staining of sorghum seed extracts. Following PAGE (5% acrylamide) the gels were incubated in a PEPC activity stain as described in Rivoal *et al*., (2002). (A) Lane 1, purified C_4 PEPC from sorghum leaf (1.0 U); lane 2, purified native C_3 PEPC from germinating sorghum seeds (Ruiz-Ballesta *et al*., 2014) (0.07 U); Lane 3 and 4, 48 and 24 h post-imbibition (0.1 and 0.06 U respectively); lane 5, dry seed (0.04 U); lane 6, stage III of development (0.08 U). (B) Lane 1, as described in panel (A); lane 2 and 3; sorghum seed extract from seeds 48 h post-imbibition (0.1 U) incubated 90 min, at 37 ºC in absence or presence of 2 µM USP-2_c (catalytic subunit of ubiquitin specific protease-2) as described in Ruiz-Ballesta *et al.* (2014).

Supplementary Figure S4. Immunopurification (IP) of PTPC (p110 and p107) from germinating sorghum seeds. Clarified extracts (CE) from 5 g of 48-h post-imbibition seeds were precleared through a pre-AminoLink column prior to their elution through an anti-COS PTPC immunoaffinity column. Pooled flow-through fractions (NR) and bound proteins (IP) that eluted from the column with 100 mM Gly-HCl (pH 2.8) were analyzed by SDS-PAGE and: (A) immunoblotting using anti-COS PTPC, or (B) total protein staining with SYPRO-Red (gel scanned using a Typhoon 8600 fluorescence imager; GE Healthcare). (A) Lanes 1 and 2 correspond to the CE and NR, respectively (10 µg of protein $lane^{-1}$); lanes 3-5 contained 20, 35, and 75 ng, respectively, of the IP eluate. (B) Lane 1, MW, protein molecular mass standards; lane 2, dephosphorylated, monoubiquitinated PTPC (RcPPC3) purified from germinating COS (30 ng) (Uhrig *et al*., 2008); lanes 3 and 4 correspond to the CE and NR, respectively (10 µg of protein $lane^{-1}$); lanes 3-5 contained 0.08, 0.2, 0.4, 0.7 µg, of the IP eluate.

Supplementary Figure S5. The p110 subunit of SbPPC2 immunopurified from germinating sorghum seeds (48 h post-imbibition) is monoubiquitinated at Lys-630. (A) TripleTOF MS/MS spectrum of the quadruply charged peptide ion of m/z 455.74. (B) TripleTOF of the triple charged peptide ion of m/z 507.92; (C) Orbitrap MS/MS spectrum of the doubly charged peptide ion at m/z 769.38; (D) Orbitrap MS/MS spectrum of the triply charged peptide ion of m/z 513.26. C- and N-terminal fragment ions are denoted by *y* and *b*, respectively. The symbol “O” on the peptide sequences represents the oxidation of methionine.

Fig. S1

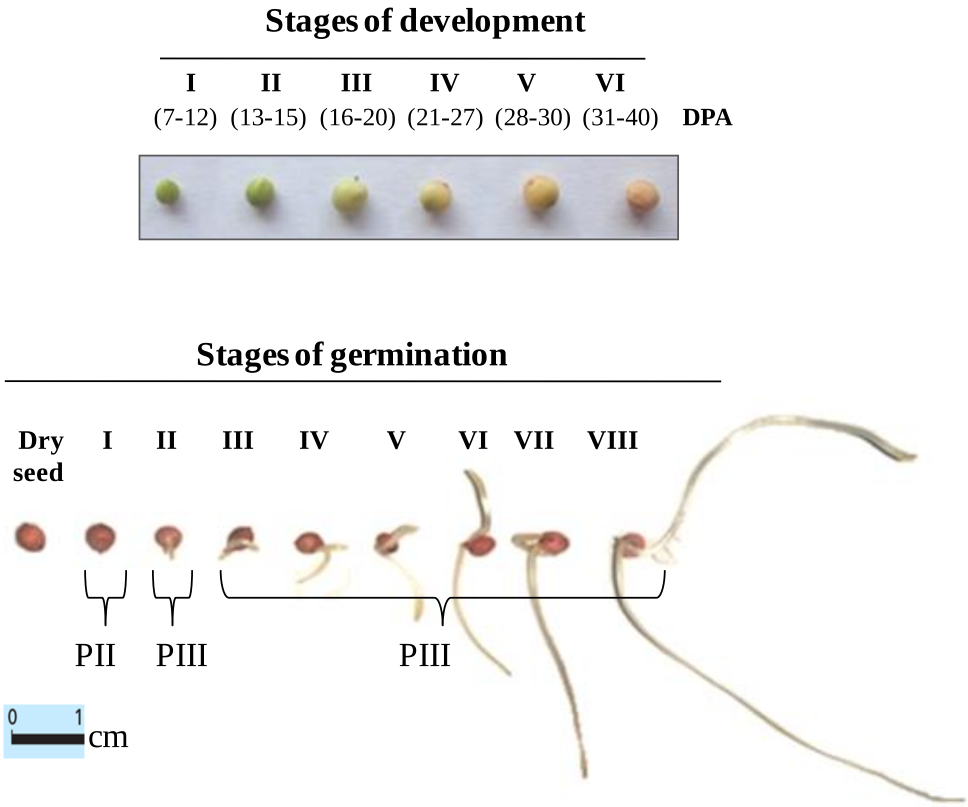
Stages of development
I II III IV V VI
(7-12) (13-15) (16-20) (21-27) (28-30) (31-40) DPA
Stages of germination
Dry seed I II III IV V VI VII VIII
PII PIII PIII
0 1 cm

Fig. S2

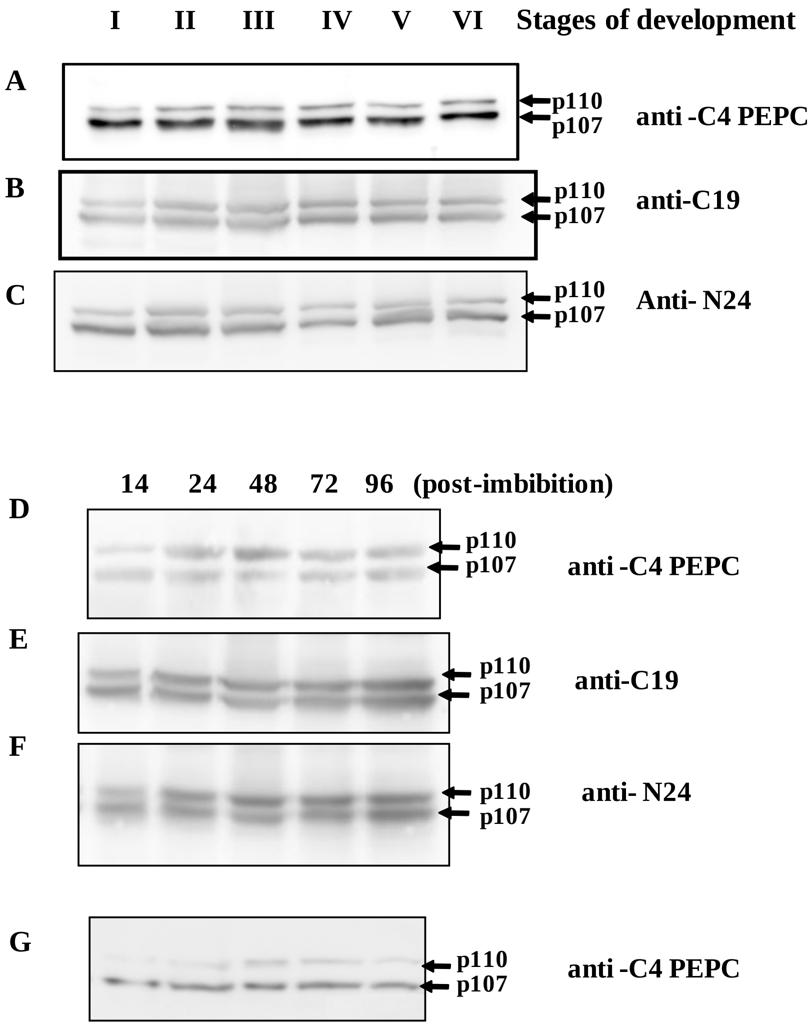
I II III IV V VI Stages of development
A
p110
p107
anti -C4 PEPC
B
p110
p107
anti-C19
C
p110
p107
Anti- N24
14 24 48 72 96 (post-imbibition)
D
p110
p107
anti -C4 PEPC
E
p110
p107
anti-C19
F
p110
p107
anti- N24
G
p110
p107
anti -C4 PEPC

S3

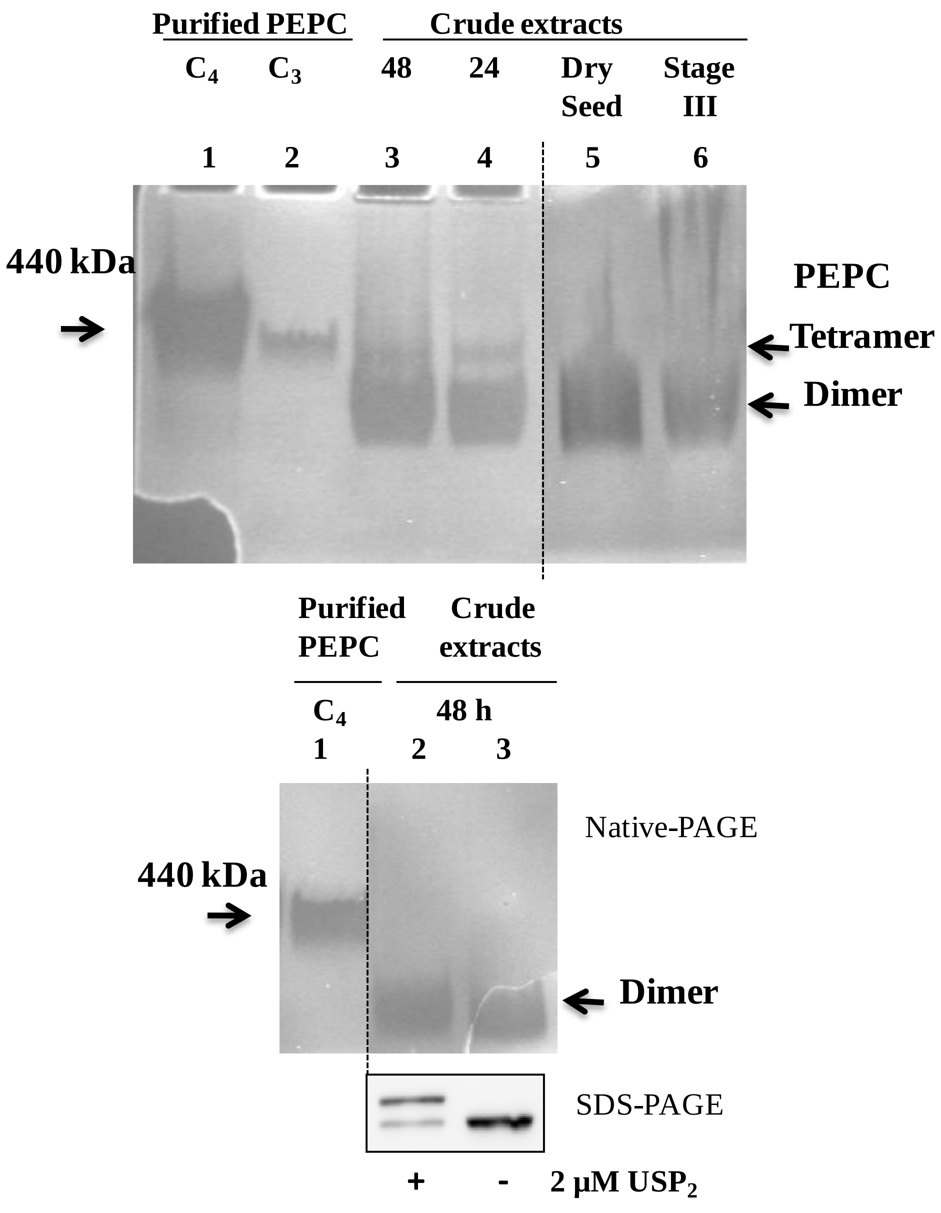
Purified PEPC
Crude extracts
C4
C3
48
24
Dry Seed
Stage III
1
2
3
4
5
6
440 kDa
PEPC
Tetramer
Dimer
Purified PEPC
Crude extracts
C4
48 h
1
2
3
Native-PAGE
440 kDa
Dimer
SDS-PAGE
+
-
2 µM USP2

S4

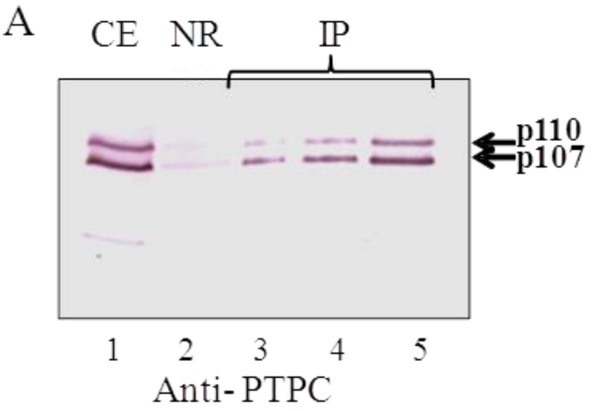
A
CE
NR
IP
p110
p107
1
2
3
4
5
Anti- PTPC

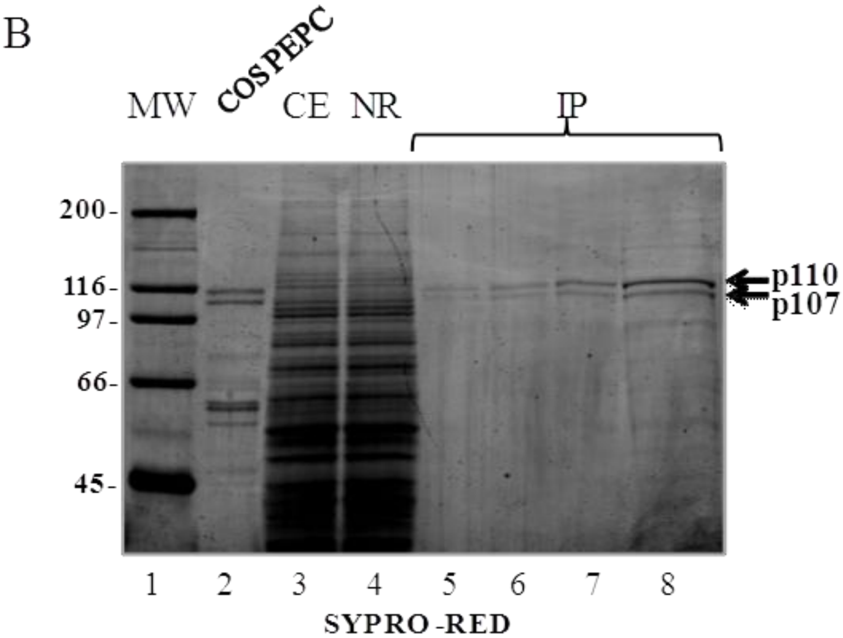
B
MW
COSPEPC
CE
NR
IP
200-
116-
97-
66-
45-
p110
p107
1
2
3
4
5
6
7
8
SYPRO -RED

Fig. S5

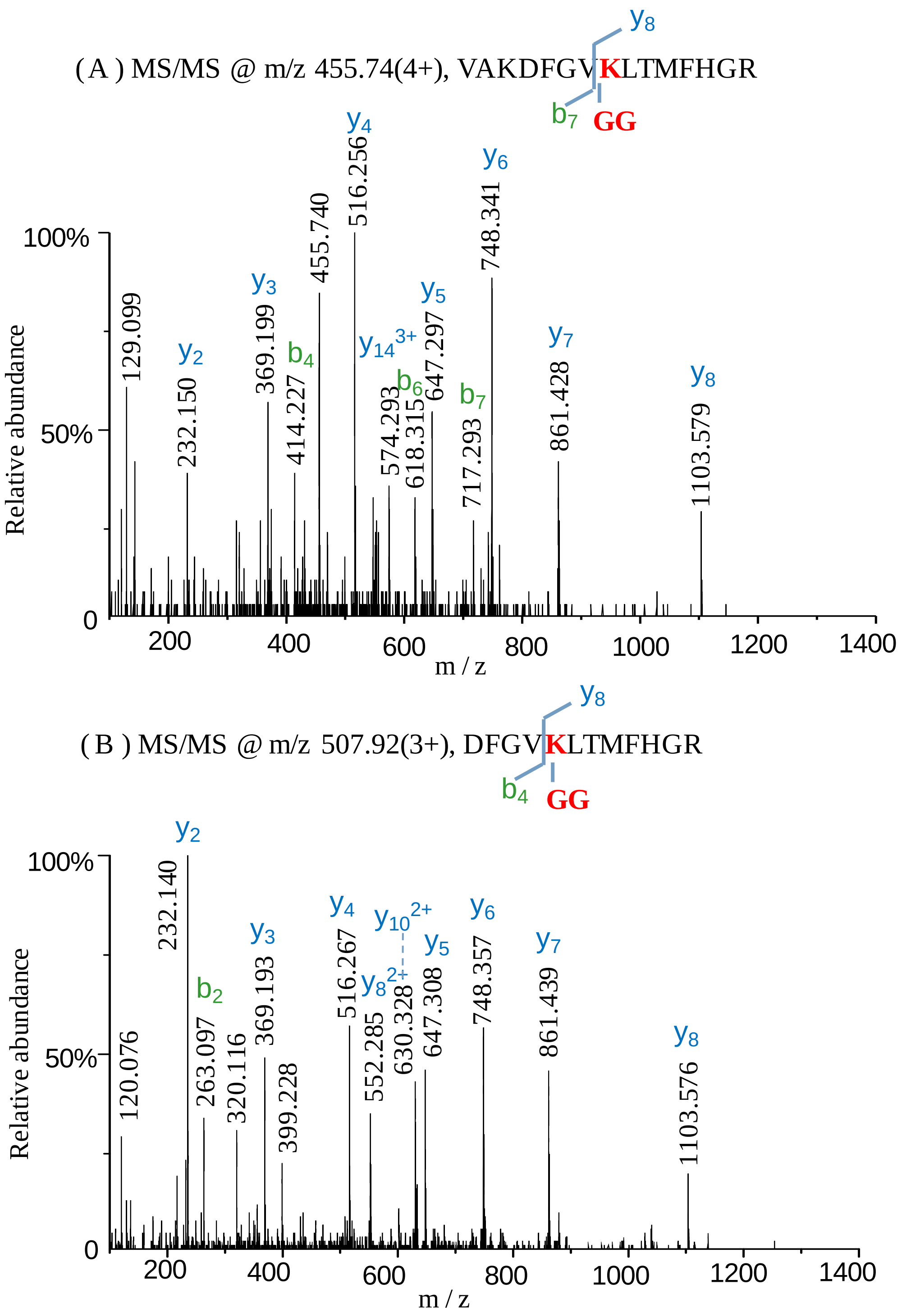
(A) MS/MS @ m/z 455.74(4+), VAKDFGVKLTMFHGR
y8
b7
GG
Relative abundance
100%
50%
0
129.099
232.150
369.199
414.227
455.740
516.256
574.293
618.315
647.297
717.293
748.341
861.428
1103.579
y2
y3
b4
y4
y14 3+
b6
y5
b7
y6
y7
y8
200
400
600
800
1000
1200
1400
m / z
(B) MS/MS @ m/z 507.92(3+), DFGVKLTMFHGR
y8
b4
GG
Relative abundance
100%
50%
0
120.076
232.140
263.097
320.116
369.193
399.228
516.267
552.285
630.328
647.308
748.357
861.439
1103.576
y2
b2
y3
y4
y10 2+
y8 2+
y5
y6
y7
y8
200
400
600
800
1000
1200
1400
m / z

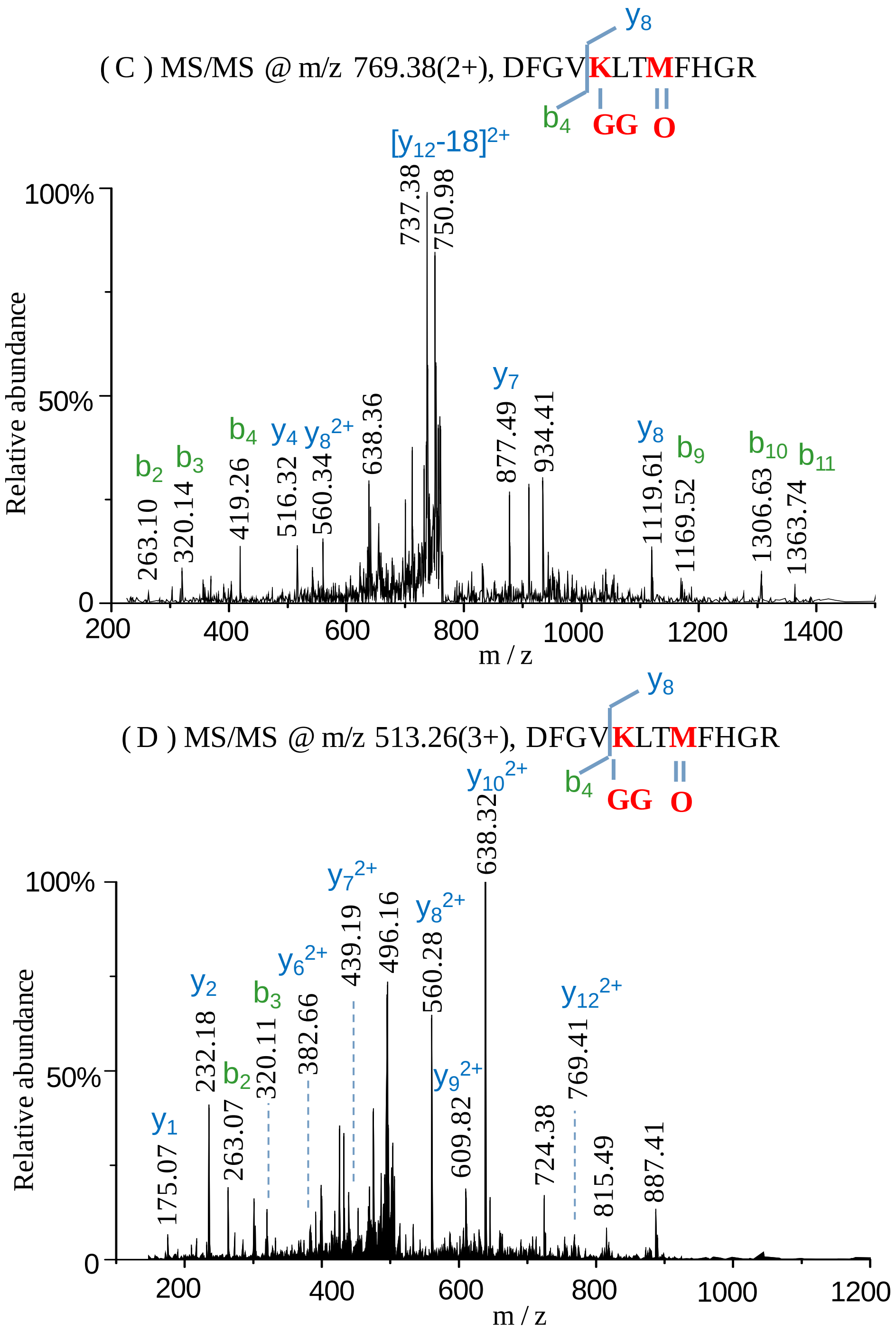
(C) MS/MS @ m/z 769.38(2+), DFGVKLTMFHGR
y8
b4
GG
O
[y12-18]2+
737.38
750.98
100%
50%
0
Relative abundance
b2
263.10
b3
320.14
b4
419.26
y4
516.32
y8 2+
560.34
638.36
y7
877.49
934.41
y8
1119.61
b9
1169.52
b10
1306.63
b11
1363.74
200
400
600
800
1000
1200
1400
m / z
(D) MS/MS @ m/z 513.26(3+), DFGVKLTMFHGR
y8
b4
GG
O
y10 2+
638.32
100%
50%
0
Relative abundance
y1
175.07
y2
232.18
b2
263.07
b3
320.11
y6 2+
382.66
y7 2+
439.19
496.16
y8 2+
560.28
y9 2+
609.82
724.38
y12 2+
769.41
815.49
887.41
200
400
600
800
1000
1200
m / z
